# Interactions between the Re-Emerging Pathogen *Corynebacterium diphtheriae* and Host Cells

**DOI:** 10.3390/ijms23063298

**Published:** 2022-03-18

**Authors:** Lisa Ott, Jens Möller, Andreas Burkovski

**Affiliations:** 1 Department of Biology, Friedrich-Alexander-Universität Erlangen-Nürnberg, Staudtstr. 5, 91058 Erlangen, Germany; lisa.baeumer@fau.de; 2 Microbiology Division, Friedrich-Alexander-Universität Erlangen-Nürnberg, Staudtstr. 5, 91058 Erlangen, Germany; jens.moeller@fau.de

**Keywords:** apoptosis, diphtheria, diphtheria toxin, mycolic acids, necrosis, pyroptosis, Shiga-like toxin

## Abstract

*Corynebacterium diphtheriae*, the etiological agent of diphtheria, is a re-emerging pathogen, responsible for several thousand deaths per year. In addition to diphtheria, systemic infections, often by non-toxigenic strains, are increasingly observed. This indicates that besides the well-studied and highly potent diphtheria toxin, various other virulence factors may influence the progression of the infection. This review focuses on the known components of *C. diphtheriae* responsible for adhesion, invasion, inflammation, and cell death, as well as on the cellular signaling pathways activated upon infection.

## 1. Introduction

*Corynebacterium diphtheriae* was first described in 1884 by Friedrich Löffler, who also showed that this bacterium is the etiological agent of diphtheria [1,2,3]. The most common form of this disease is respiratory diphtheria [4], which is characterized by mild fever and an exudative pharyngitis at the beginning of infection. During progression of the infection, a greyish white pseudo-membrane may be formed on the tonsils, pharynx, and larynx, composed of fibrin and secreted by the damaged nasopharyngeal epithelia, destroyed host cells, and colonizing bacteria. Detachment of the pseudo-membrane by coughing may cause bleeding of the epithelial tissue, and subsequently, decaying erythrocytes may stain the pseudo-membrane a dirty brownish color. Extension of the inflammation into the nasal cavity and larynx may cause an obstruction of the airways, resulting in dyspnea up to suffocation and death [5].

**Figure 1:**
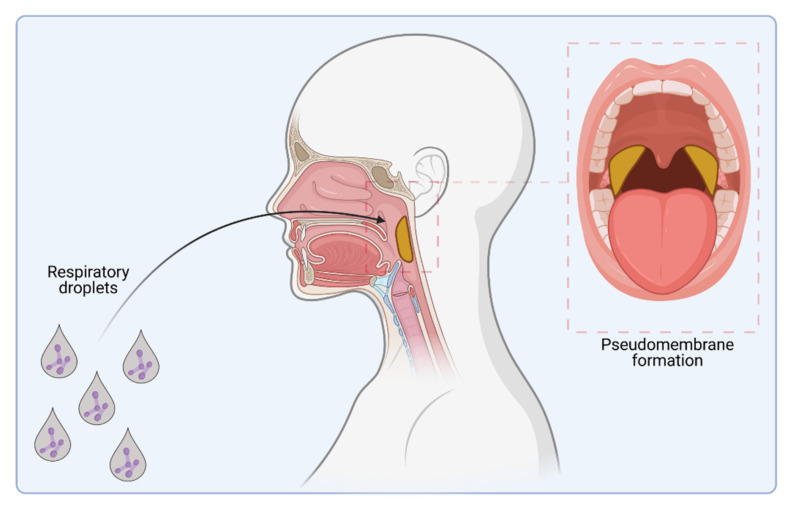
*C. diphtheriae* infection. Infection pathway by respiratory droplets and pseudo-membrane formation (indicated in yellow) caused by colonization of the upper respiratory tract (figure created with BioRender.com).

Classical diphtheria of the upper respiratory tract is spread from person to person by respiratory droplets (Figure 1). Additionally, other secretions and contaminated materials may be sources of infection, especially in the case of cutaneous diphtheria, where wounds or insect bites are the typical entry sites [5,6].

Before introduction of mass vaccination, children were the main victims of diphtheria, which indicates that *C. diphtheriae* was widely disseminated among the population, leading to early contact with the pathogen. With the beginning of industrialization and urbanization, diphtheria became more prevalent and developed into a leading cause of infant mortality. Up to four fifths of children infected with diphtheria died [7].

As the detrimental action of diphtheria toxin is the main cause of the often-fatal outcome of infection, it was a prime target to combat respiratory diphtheria. Emil von Behring was the first person to develop a treatment of diphtheria based on the administration of diphtheria antitoxin produced by horses [8], for which he was honored with the first Nobel Prize for Medicine in 1901 [9]. The first toxoid vaccine was produced by Ramon in 1923 by formalin treatment of diphtheria toxin, and it was the basis of subsequent mass vaccination starting in industrialized countries in the 1920s [10]. After implementation of the World Health Organization’s Expanded Programme on Immunization (EPI) in 1974, only relatively small and local outbreaks occurred until the 1990s [11]. This changed with the breakdown of the former Union of Socialist Soviet Republics, when a large-scale outbreak leading to a diphtheria pandemic between 1990 and 1998 occurred, with more than 157,000 reported cases and over 5000 deaths [12,13,14,15]. This pandemic was finally stopped by mass immunization, especially of adults with waning antibody levels. Despite continuing global efforts and stable vaccination coverage, diphtheria is not eradicated today. Between 2015 and 2019, diphtheria outbreaks occurred, for example, in Bangladesh, Haiti, South Africa, Venezuela, and Yemen [4,16,17,18,19,20], and, moreover, the worldwide number of reported cases of diphtheria has increased within the last few years [21,22] (Table 1).

In consequence, today, *C. diphtheriae* is not only considered as a re-emerging pathogen [23,24,25], but still one of the most important global pathogens [26,27]. It can be expected that the global SARS-CoV-2 pandemic, which overwhelmed public health systems in many countries, will result in a further increase of diphtheria due to neglected vaccination programs, and also recent military conflicts such as in Yemen, Ethiopia, or Ukraine may interfere with vaccination. Furthermore, antibiotic-resistant strains of *C. diphtheriae* are increasingly observed [28,29]. Consequently, not only surveillance of cases, but also continuing research focusing on the re-emerging pathogen is crucial. In fact, the interaction of *C. diphtheriae* with host cells turned out to be much more complex than initially expected when *C. diphtheriae* was considered as extracellular pathogen. The manuscript presented here summarizes our knowledge on *C. diphtheriae*–host interaction on a molecular level.

## 2. Host Cell Binding Properties of *C. diphtheriae*

**Figure 2:**
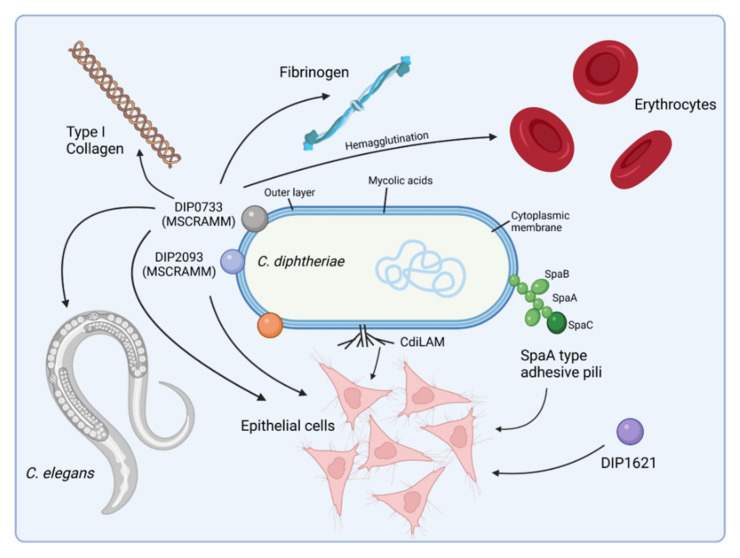
Adhesion of *C. diphtheriae*: a multi-factorial process. *C. diphtheriae* can bind different epithelial cell types in a strain-specific manner. Several proteins involved in this process have been identified thus far, including adhesive pili and MSCRAMMS (Microbial Surface Components Recognizing Adhesive Matrix Molecules), which mediate attachment to fibrinogen or collagen. Deletion or disruption of single genes encoding one of these proteins results typically in only a minor loss of adhesion, indicating that a combination of independent adhesion mechanisms act together. In addition, *C. diphtheriae* can bind to human erythrocytes, which may support spreading of the bacteria via the bloodstream within the whole body [31] (figure created with BioRender.com).

To recognize and colonize host cells, pathogens display molecules at their surface, e.g., cell wall-linked surface proteins and fibrous protein polymers such as fimbriae and pili that bind to specific receptors on the host cell and often trigger immune responses of the host. In the case of *C. diphtheriae*, several molecules that play a pivotal role in adhesion of *C. diphtheriae* were characterized, and as in the case of the closely related *Mycobacterium tuberculosis*, adhesion of *C. diphtheriae* to the host cell is a multifactorial process [30,31] (Figure 2).

Mutant analyses revealed that *C. diphtheriae* type-strain NCTC13129 is able to assemble different types of pili on its surface, which are important for bacterium–host cell contact and host cell preference [32,33,34,35]. Furthermore, it has been shown that the expression of different pilin subunit proteins influences the pili length in *C. diphtheriae* [33,36]. Three pili clusters are known for *C. diphtheriae* NCTC13129—*spaABC*, *spaDEF*, and *spaGH.* Later studies characterized 42 clinical isolates regarding toxigenicity and pili expression by PCR and immunoblotting against different pili subunits and found that presence of pilus-encoding genes varies considerably between different wild-type strains and the SpaA-type is the pilus most represented in the investigated strains [37]. By investigating the correlation between pilus expression and adhesion efficiency, it turned out that *C. diphtheriae* wild-type strains differ in pili formation. For example, strain ISS4060 completely lacks pili structures, while strain ISS3319 possesses spike-like pili. Remarkably, both strains showed comparable adhesion rates, which indicates that pili formation and adhesion are not strictly coupled [38]. In summary, pili of *C. diphtheriae* may help the bacteria to bind to host cells, but they are not mandatory for adherence, since further proteins contribute to this process. No receptor for corynebacterial pili was identified experimentally, while it was shown for *M. tuberculosis* that pili contribute to the binding of laminin present in the extracellular matrix of the host cells [30]. 

In addition to pili, MSCRAMMs (Microbial Surface Components Recognizing Adhesive Matrix Molecules) are widely distributed in Gram-positive pathogenic bacteria [39] including *C. diphtheriae*. Up until now, two proteins were shown to play an important role in adherence of *C. diphtheriae* to the extracellular matrix (ECM) of eukaryotic cells. *C. diphtheriae* protein DIP0733 can interact with collagen, fibrinogen, erythrocytes, and epithelial cells and is essential for colonization of *Caenorhabditis elegans* [40], while DIP2093 is involved in binding to type I collagen, adherence to epithelial cells, and in causing severe inflammatory response in the host [41]. Since DIP0733 and DIP2093 are not only acting as MSCRAMMs but also bind to epithelial cells and are involved in colonization of nematodes, the question arises as to which receptors are recognized by them. 

This is also the case for the *C. diphtheriae* proteins DIP1281, DIP1546, and DIP1621. DIP1281, previously annotated as hypothetical invasion-associated protein, was one of the first functional characterized proteins with respect to *C. diphtheriae* adhesion and invasion [42]. A DIP1281 mutant was generated in two non-toxigenic isolates, ISS3319 and ISS4060, and further characterized by ultrastructural analysis of the surface and in host–pathogen interaction studies. The results obtained hint to a more general function of DIP1281. The protein seems to be involved in cell surface organization of the bacterium, and due to re-organization of the cell surface in mutant strains, adhesion and invasion of host cells seems to be inhibited [42]. 

DIP1546 was identified in a Tn*5* transposon screen and showed highly reduced adherence to Detroit562 cells and impaired colonization of *C. elegans* [43]. Further data with respect to interaction with host cells are not available.

DIP1621 was identified in a Tn*5* transposon mutant pool of *C. diphtheriae* strain 225 that was screened for reduced adherence to HEp-2 cells. The corresponding mutant strain showed an adhesion rate of only 15.2% compared to the wild type [44], indicating that this protein is a major part of a complex system of adhesins. 

In addition to the proteins mentioned above, non-proteinacous compounds also seem to be involved in adhesion. A lipoarabinomannan variant isolated from *C. diphtheriae* and designated CdiLAM was isolated, which supports adhesion of *C. diphtheriae* to human respiratory epithelial cells, but in contrast to DIP0733 did not function as hemagglutinin to human erythrocytes [45].

## 3. Invasion of *C. diphtheriae*—What We Know to Date

*C. diphtheriae* was originally thought to be an exclusively extracellular pathogen of the respiratory tract. However, various studies have shown that *C. diphtheriae* may also cause systemic infections such as bacteremia, endocarditis, septic arthritis, and osteomyelitis, and thus must be able to gain access to deeper tissues [46,47,48,49,50]. Furthermore, it was shown that the bacteria are also able to invade host cells. Although infections of deeper pats of the body and invasion of host cells are in principle different processes, some multifunctional proteins of *C. diphtheriae* are involved in both processes. 

To date, three proteins—DIP0733, DIP2093, and CDCE8392_081—are known to play a major role in host cell invasion by *C. diphtheriae* and establishment of the bacteria in the body [40,41,51,52]. 

Sabbadini and co-workers started to elucidate the role of DIP0733 when they identified the proteins responsible for two prominent bands in SDS-PAGE and Western blotting experiments, designated 67-72p on the basis of their apparent molecular mass [51]. For this purpose, 67-72p was purified by ammonium sulfate precipitation from toxigenic strain CDC-E8392. Cytoskeletal changes with accumulation of polymerized actin in HEp-2 cells beneath adherent 67-72p-adsorbed latex beads were observed by actin fluorescence staining. Additionally, 3-(4,5-dimethylthiazol-2-yl)-2,5-diphenyl-2H-tetrazolium bromide (MTT) assays revealed a significant decrease in viability of HEp-2 cells treated with 67-72p. A variety of morphological changes were observed in HEp-2 cells after treatment with 67-72p, including vacuolization, nuclear fragmentation, and the formation of apoptotic bodies. Sabbadini and co-workers concluded that DIP0733 may be directly involved in bacterial invasion and apoptosis of epithelial cells in the early stages of diphtheria and invasive infection by *C. diphtheriae* [51]. Antunes and co-workers supported these findings by generating a DIP0733 mutant strain, which was strongly decreased in its ability to adhere and invade epithelial cells. In addition, on the basis of its fibrinogen-binding activity, DIP0733 may play a role in avoiding recognition of *C. diphtheriae* by the immune system [40]. In conclusion, DIP0733 seems to be essential for *C. diphtheriae* strain CDC-E8392 to adhere to and invade host cells. By binding ECM, the bacteria may hide from the host immune system and can penetrate deeper tissues. Moreover, if it has gained access into the blood vessels, it can spread through hemagglutination via the erythrocytes in the entire bloodstream. 

DIP2093, previously annotated as putative adhesin of the serine-aspartate repeat (Sdr) protein family, binding the extracellular matrix surrounding eukaryotic cells, was characterized with respect to invasive properties by Peixoto and co-workers [41]. A DIP2093 mutant revealed significantly lower adhesion and invasion rates on epithelial cells, strongly reduced numbers of viable bacteria in mouse macrophages, and was less harmful toward the nematode *C. elegans* when compared to the wild-type NCTC13129 [41]. Furthermore, a DIP2093 mutant was strongly attenuated in causing clinical signs of arthritis in mice [41], implicating a major role of DIP2093 in invasive infections by *C. diphtheriae*. 

Some bacterial species are naturally resistant towards tellurite (TeO_3_^2−^), the oxidized and soluble form of tellurium (Te), a naturally occurring trace element that is toxic to pro- and eukaryotes. The most well-known tellurite-resistant (Te^R^) pathogen is *C. diphtheriae* [53]. The molecular mechanism behind this phenomenon is not yet fully understood, but the presence of Te^R^ determinants in pathogenic bacteria suggests that these genes might provide some selective advantage in the environment and may also contribute to pathogenicity [54,55]. 

Dos Santos and co-workers identified a putative Te^R^ determinant in *C. diphtheriae* strain CDC-E8392 (CDCE8392_0813, hypothetical protein included in the TeO_3_^2−^ resistance/dicarboxylate transporter family) by in silico analyses [52]. A significant increase in susceptibility to TeO_3_^2−^ was observed for a corresponding mutant strain as well as reduced ability to survive within Hep-2 cells. Furthermore, the mutant showed less detrimental effects to *C. elegans*. Interestingly, this protein seems to be essential for intracellular survival in the host but not for adhesion since the mutant did not show any changes in hemagglutination and adherence to hydrophobic surfaces or epithelial cells compared to the wild type [52]. 

Tellurite resistance is discussed as being connected to oxidative stress response. Interestingly, gene disruption of *oxyR*, encoding the global oxygen regulator *C. diphtheriae*, affected adherence patterns, invasion, and intracellular survival in epithelial cells, as well as the arthritogenic potential of *C. diphtheriae* in mice [56]. The exact mechanisms behind these effects are unclear.

In summary, *C. diphtheriae* enters the host via open wounds and is able to spread in the body via the bloodstream and reach deeper tissues and organs. As an opportunistic intracellular pathogen, *C. diphtheriae* can cause severe infections independent of the toxin. The way in which the bacteria penetrate the cells, for which receptors are responsible for the uptake of the bacteria and how they survive within the host cell, remains an exciting question to be solved. Table 2 summarizes the current knowledge of corynebacterial proteins that are involved in the host–pathogen interaction as well as their putative receptors on the host cell.

## 4. Inflammatory Signaling in Response to *C. diphtheriae* Infection

**Figure 3:**
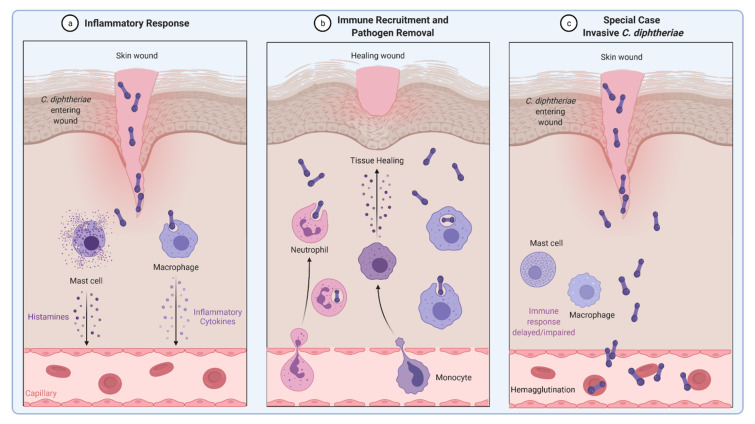
Inflammatory response induced by *C. diphtheriae*. (**a**,**b**) Inflammatory response caused by non-invasive *C. diphtheriae*. Entering bacteria lead to recruitment of immune cells such as neutrophils and macrophages and removal of the pathogen. (**c**) Invasive *C. diphtheriae* remain undetected by the host immune system through unknown mechanisms, gain access to deeper tissues and blood vessels, and spread through the whole body by binding erythrocytes (hemagglutination) (figure created with BioRender.com).

The common way of a eukaryotic cell to react to an infection with pathogenic microorganisms is an inflammatory response. Secretion of inflammatory cytokines and histamines leads to recruitment of immune cells such as neutrophils and natural killer cells that help to engulf and remove the pathogen (Figure 3a,b). Obviously, *C. diphtheriae* has developed mechanisms to avoid recognition by the host immune system and allow the bacteria to persist within host cells, proliferate, and enter the blood vessels, where they can bind to erythrocytes (hemagglutination) and spread through the whole body via the bloodstream (Figure 3c).

To unravel the reaction of host cells triggered by *C. diphtheriae*, HeLa NFκ-B reporter cells were infected with six non-toxigenic isolates and one toxin-encoding strain to monitor the response of human host cells to *C. diphtheriae* infection. A combination of adhesion and invasion assays was used and compared with NFκ-B induction measured by luciferase reporter activity of the cells [66]. The results indicated that internalization of the bacteria is crucial for NFκ-B induction, while adhesion to the host cell had no effect. These data were supported by fluorescence microscopy assays proving translocation of p65 protein into the nucleus, which is a hallmark of the NFκ-B pathway [66]. P65 translocation only occurred in combination with invading *C. diphtheriae* strains. Tetracycline-inactivated bacteria were still able to adhere to host cells but were not found inside the epithelial cells and were not able to induce the NFκ-B pathway [66]. Obviously, invasion is an active process of the bacteria, and only inside the cell may bacteria or their structures be recognized by the host, resulting in activation of the NFκ-B pathway. 

A prominent candidate for a bacterial structure, which may be recognized by the host, is the complex cell wall of *C. diphtheriae*. As in case of almost all other members of the CMNR (*Corynebacterium*, *Mycobacterium*, *Nocardia*, *Rhodococcus*) group, *C. diphtheriae* has a mycolic acid layer, which is functionally equivalent to the outer membrane of Gram-negative bacteria [67,68]. 

Using organic solvents, lipids were extracted from the mycomembrane and further analyzed [57]. Plate-bound lipid extracts of *C. diphtheriae* and several other *Corynebacterium* species bound to Mincle-Fc in a dose-dependent manner. Additionally, these plate-bound corynebacterial glycolipids as well as heat-killed bacteria were able to induce granulocyte colony-stimulating factor (G-CSF) and nitrite production in bone marrow-derived macrophages (BMM). Furthermore, it was shown that Mincle and its adaptor protein FcRγ is required for immune response of BMM [57]. Toll-like receptor 2 (TLR2) is essential for macrophage activation by glycolipids and heat-killed corynebacteria. Additionally, it can be concluded that TLR2 is responsible for upregulation of Mincle expression in response to contact with corynebacterial glycolipids [57], which was also shown for the mycobacterial cord factor trehalosyl-dimycolate [69].

Recently, a combination of BMMs and the human monocytic cell line THP-1 was used for infection with a panel of seven non-toxigenic and one toxigenic strain DSM43989. Additionally, the non-pathogenic strain *C. glutamicum* ATCC13032 served as a control. In this case, the toxigenic strain showed lowest amount of intracellular colony-forming units (CFUs), indicating that the bacteria were not taken up by the cell. This result was supported by application of Toll-like receptor 9 (TLR9) reporter cells. TLR9 is a receptor that is expressed in the endoplasmatic reticulum and located in endolysosomal compartments and detects CpG unmethylated DNA. Almost no TLR9 activation was detectable by strain DSM43989 [58]. Interestingly, although the non-pathogenic strain *C. glutamicum* showed no viable CFUs independent of the host cell, this strain was detected by TLR9, indicating that these bacteria were degraded immediately after endocytosis. Additionally, when pro-inflammatory cytokine production was measured, all *C. diphtheriae* strains led to higher G-CSF production in comparison to interleukin-6, while the non-pathogenic strain *C. glutamicum* showed strongly reduced cytokine production [58]. 

**Figure 4:**
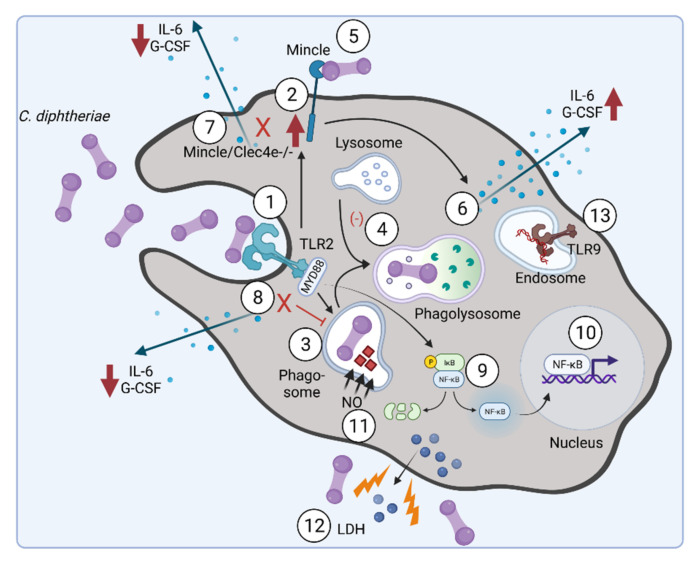
*C. diphtheriae* recognition by macrophages. Binding of *C. diphtheriae* by TLR2 (1) leads on the one hand to upregulation of the C-type lectin receptor Mincle (2) and on the other hand to phagocytosis of the bacteria (3), resulting in phagosome–lysosome fusion, which is somehow delayed by *C. diphtheriae* (4). Furthermore, binding of *C. diphtheriae* to Mincle (5) triggers the production of pro-inflammatory cytokines (6), which was confirmed by reduced cytokine production in Clec4e-deficient cells (7). Additionally, in Myd88-deficient cells the cytokine production as well as the uptake of the bacteria was completely blocked (8). Further signs of inflammation caused by pathogenic corynebacteria are the activation of NFκ-B-signaling (9), resulting in upregulation of pro-inflammatory genes (10), and the production of nitric oxide (NO) (11). In the case of the infection of THP-1 cells, a cytotoxic effect of *C. diphtheriae* was detectable by LDH release (12). TLR-9 activation can be observed for non-toxigenic strains (13) [58] (figure created with BioRender.com).

In conclusion, our results indicate that the TLR2/Myd88 pathway is crucial for phagocytosis of the bacteria and upregulation of the CLR Mincle. Furthermore, infection of THP-1 cells with *C. diphtheriae* led to strain-specific phagocytosis of the bacteria [58], in the same manner as was observed in former studies, when human HeLa and Detroit562 cell lines were infected [66]. Figure 4 summarizes the current knowledge about *C. diphtheriae*–macrophage interaction.

## 5. *C. diphtheriae*-Induced Apoptosis and Necrosis

Diphtheria toxin is most likely the best studied bacterial toxin [64,70]. In 1888 Roux and Yersin proved that diphtheria toxin is responsible for often fatal damages on organs such as heart and kidneys when they injected sterilized *C. diphtheriae* culture supernatants to guinea pigs, which developed symptoms similar to those observed cases of diphtheria patients [65]. The toxin is encoded by a β-corynebacteriophage, which is able to lysogenize *C. diphtheriae* and its closely related relatives *Corynebacterium pseudotuberculosis* and *Corynebacterium ulcerans* [71,72]. The *tox* gene is under control of the transcriptional regulator DtxR. When Fe^2+^ is available, DtxR binds to the *tox* operator and blocks transcription. Vice versa, DtxR is inhibited by low iron concentrations, leading to transcription of the *tox* gene [73].

Diphtheria toxin is synthesized as a precursor protein with a 25 amino acid signal sequence and is extracellularly secreted as a single polypeptide chain of 535 amino acids. The extracellular protein has a molecular weight of 62 kDa and contains three domains: the N-terminal catalytic domain (C or FA-domain) and fragment B (FB or carboxy-terminal receptor-binding R-domain), which are linked by a disulfide bond, and the translocation T-domain [64,74,75]. The ADP ribosytransferase activity of the catalytic domain of the toxin is activated by proteolytic cleavage of the α-carbon backbone at Arg193, which is located in a loop formed by a disulfide bond between Cys186 and Cys201 [70].

**Figure 5:**
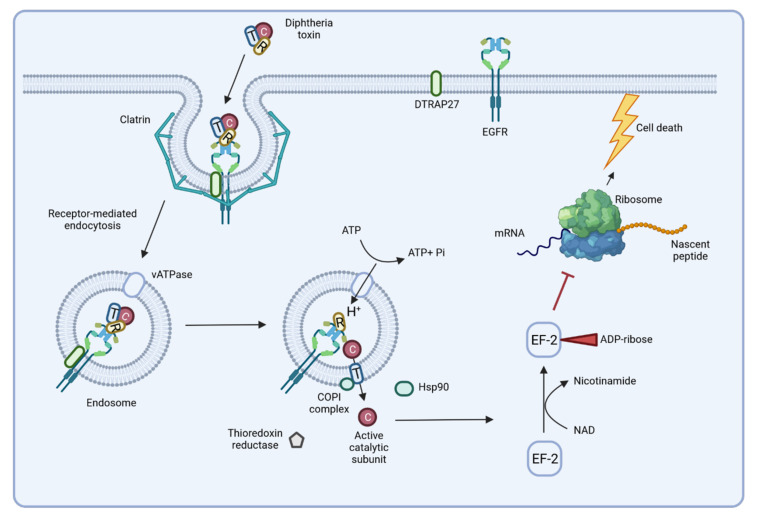
Delivery and action of diphtheria toxin. The B-subunit of the toxin binds to the host receptor HB-EGF, leading to receptor-mediated endocytosis. Once in the endosome, acidification of the lumen induces pore formation, and the catalytic domain of the toxin is released into the cytoplasm. ADP ribosyltransferase activity of the catalytic domain inactivates elongation factor 2 (EF-2) and protein biosynthesis stops, inducing cell death by apoptosis (see below) (figure created with BioRender.com).

In the un-cleaved form, the toxin is inactive and may be distributed to different organs when secreted into the bloodstream. The subsequent delivery of DT to the cytosol of a target cell depends on binding to the toxin receptor HB-EGF of the host cell, which is supported by the diphtheria toxin receptor-associated protein 27 (DTRAP 27) [70,76]. Receptor-mediated endocytosis of the complex by the host cell is followed by an acidification of the endosome due to the activity of a vacuolar (v)ATPase [70]. Acidification induces unfolding of the translocation domain and its insertion into the endosomal membrane, where it forms a pore with a diameter of approximately 20 Å [77,78]. After cleavage of the toxin, the catalytic domain is released into the cytosol through the pore of the translocation domain. The translocation process involves cellular proteins such as the COPI complex and a cytoplasmic thioredoxin reductase [70], while refolding of the catalytic domain and activation of its ADP ribosyltransferase activity is supported by the Hsp90 chaperone. Subsequently, the catalytic domain ADP ribosylates elongation factor 2 (EF-2) of the host cell. This leads to an inhibition of the protein synthesis by the ribosome and apoptosis is induced [74,79] (Figure 5). A single toxin molecule is sufficient to stop protein synthesis of a cell and a toxin concentration of 0.1 μg per kg body weight is lethal for humans [9,75].

Interestingly, a *C. diphtheriae* strain was detected, which showed severe detrimental effects without being toxigenic. HC04 was isolated from a catheter of a 7-year-old girl that developed complications including arthritis, myositis, and peripheral and central nervous system emboli, as well as a microaneurysm with brain hemorrhage, and died due to septic shock caused by endocarditis [80,81]. Infection of THP-1 cells with *C. diphtheriae* HC04 resulted in condensation of DNA in macrophage nuclei and induced cell lysis, which are clear signs of necrosis [82]. Live cell imaging experiments revealed that these detrimental effects on macrophages are due to bacterial endocytosis and replication within the host cells. By using flow cytometry analyses with annexin V as a FITC 54 conjugate in combination with propidium iodide, a phosphatidyl serine (PS) exposure of the cells was identified and plasma membrane damage was detected [83]. Additionally, upon infection with *C. diphtheriae* HC04, several stages of cell death and associated changes such as viable, stressed, early/late apoptosis, and primary/secondary necrosis have been distinguished [83]. The probable reason for this high virulence potential was recently identified as a new cytotoxic protein annotated as putative ribosome-binding protein (Rbp) [61]. The corresponding Rbp mutant was tested in a combination of invertebrate in vivo infection model systems, *C. elegans* and *G. mellonella* model systems, and various in vitro animal and human cell line assays. Highly detrimental effects were observed, depending on the presence of Rbp in this study [61]. 

Besides HCO4, another *C. diphtheriae* strain, CDC-E8392, has already been described to induce apoptosis in host cells, with DIP0733 as a major factor involved in this process [51]. In summary, Rbp of strain HC04 and DIP0733 detected in CDC-E8392 are multifactorial proteins with high virulence potential, especially regarding induced cell death in host cells.

**Figure 6:**
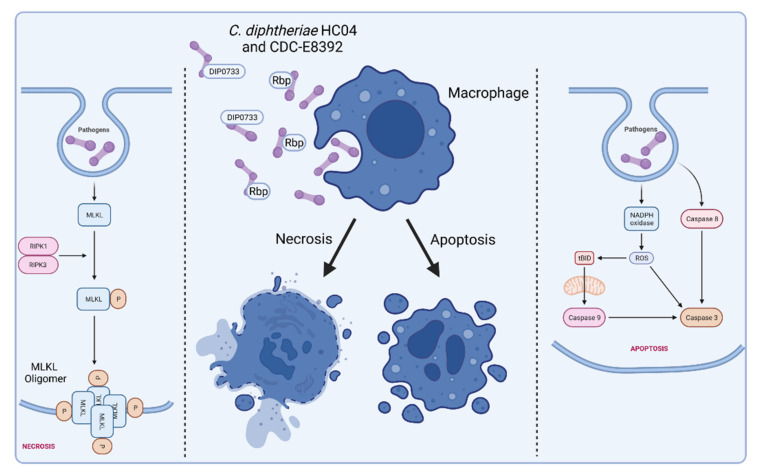
*C. diphtheriae*-induced necrosis and apoptosis in macrophages. Infection of human macrophage cell lines leads to induction of necrosis and apoptosis. Factors that are involved in these processes are Rbp from strain HCO4 and DIP0733 from CDC-E8392. The molecular mechanisms by which these proteins act is unclear thus far, and more detailed biochemical analyses are required to understand the cytotoxic activity. Necrosis is highly regulated by cellular processes that are characterized by a loss of cell membrane integrity, intracellular organelles, and cell swelling [84,85]. In contrast to apoptosis, necrosis represents a form of cell death that is optimally induced when caspases are inhibited [86,87,88]. Regulated or programmed necrosis eventually leads to cell lysis and release of cytoplasmic content into the extracellular region that often results in tissue damage and intensive inflammatory response. Apoptosis is characterized by nuclear chromatin condensation, cytoplasmic shrinking, dilated endoplasmic reticulum, and membrane blebbing [89]. Apoptosis is considered as controlled suicide of the cell, which, in contrast to necrosis, does not include the release of cell plasma and thus does not trigger an inflammatory reaction. (Receptor interacting protein kinase 1 and 2 (RIPK1, RIPK2), mixed lineage kinase domain-like (MLKL), reactive oxygen species (ROS), membrane targeted death ligand (tBID); figure created with BioRender.com).

Figure 6 illustrates the *C. diphtheriae*-induced necrosis and apoptosis in macrophages by *C. diphtheriae* strains HCO4 and CDC-E8392. These cell death mechanisms may protect *C. diphtheriae* against destruction by macrophages and support dissemination via the bloodstream (Figure 3).

## 6. Inflammasome Activation and Pyroptosis

In addition to necrosis and apoptosis, pyroptosis is described as another form of programed cell death. As in case of apoptosis, chromatin condensation is observed during pyroptosis, but in contrast apoptosis, when the nucleus breaks up into multiple chromatin bodies, in pyroptosis, the cell nucleus remains intact. Pyroptosis is a caspase-1-dependent type of cell death that is mediated by the cleavage of gasdermin D and the subsequent formation of pores in the cell membrane leading to the release of cytoplasmic content into the extracellular space [90]. So-called inflammasomes are involved in the activation of caspase-1 and the maturation of interleukin IL-1β and IL-18, which are mainly released via gasdermin D pores. Pyroptosis is seen primarily in inflammatory cells such as macrophages and may be trigged by bacterial infections [91]. Recent studies of Ott and co-workers investigated caspase-1-dependent inflammasome activation by corynebacteria in human macrophages. THP-1 caspase-1-deficient cells were infected with viable and dead *C. glutamicum* ATCC13129 and different *C. diphtheriae* isolates at MOI 1 and 10 (Ott et al., unpublished). After incubation, the supernatant was transferred to IL-1β sensor cells in order to monitor bioactive IL-1β released by test cells upon inflammasome activation. When THP-Null cells, which served as control, were infected, dead bacteria of all strains led to IL-1β release in a dose-dependent manner. Interestingly, viable bacteria of strains ATCC13129, ISS4060, ISS4746, ISS4749, and DSM43989 led to IL-1β secretion in THP-1-Null cells, while ISS3319, DSM43989, DSM44123, and INCA-402 did not. In case of THP-1 caspase-1 deficient cells, the results became more complex. Dead bacteria of all strains did not lead to IL-1β release in these cells anymore, indicating that the IL-1β secretion induced by dead bacteria is caspase-1-dependent. Remarkably, viable bacteria of strains ATCC13129, ISS4060, ISS4746, ISS4749, and DSM43988 were still able to trigger IL-1β secretion in THP-1 caspase-1-deficient cells, indicating there is a strain-specific caspase-1-independent inflammasome activation by corynebacteria (Ott and co-workers, unpublished). The fact that only living bacteria of some strains induced caspase-1-independent signaling suggests active secretion of the responsible effectors.

**Figure 7:**
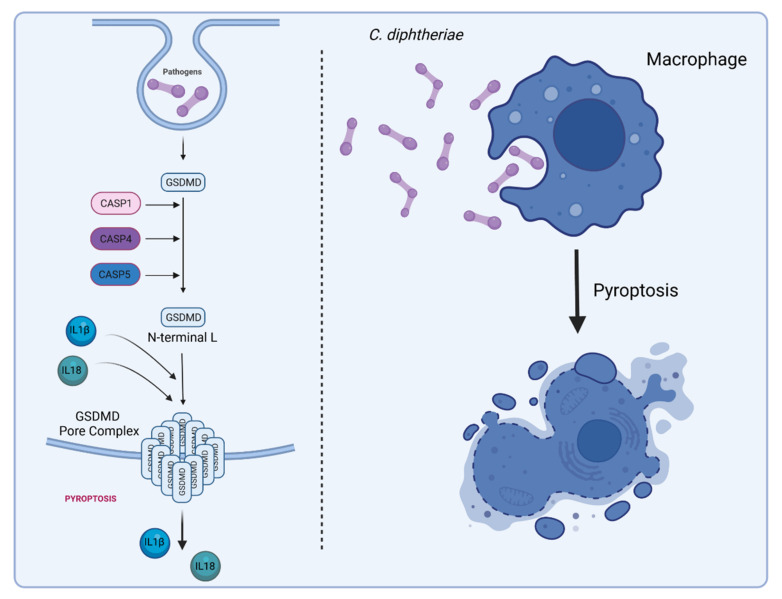
*C. diphtheria*-induced pyroptosis in macrophages. Gasdermin D (GSDMD) serves as a specific substrate of caspase-1, -4, -5 (in humans), and -11 (in mice) and as an effector molecule for the lytic and highly inflammatory form of pyroptosis [95,96]. The pore-forming activity of the N-terminal cleavage product causes cell swelling and lysis to prevent intracellular pathogens from replicating, leading to the release of cytoplasmic content such as the inflammatory cytokines IL-1β and IL-18 into the extracellular space to recruit and activate immune cells to the site of infection [97] (figure created with BioRender.com).

**Figure 8:**
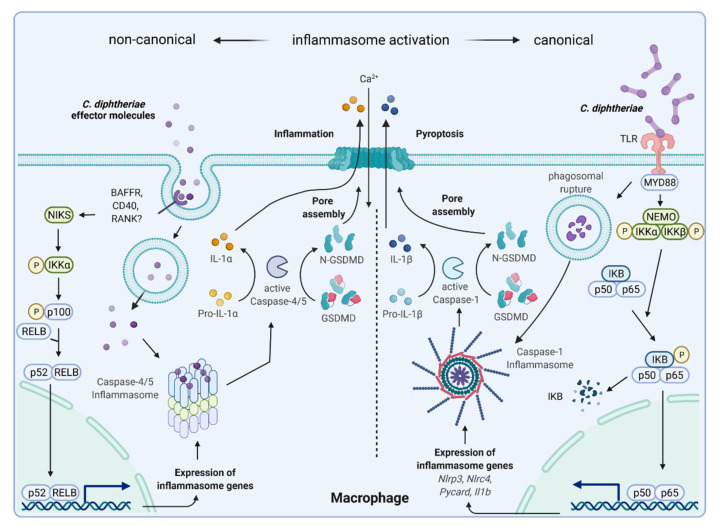
Hypothetical *C. diphtheriae*-induced non-canonical and canonical inflammasome activation and pyroptosis. Inflammasomes are multimeric proteins that play a pivotal role in host defense against invading pathogens. Canonical inflammasomes such as NLRP3 respond to a wide range of PAMPs and DAMPs and their activation in macrophages requires two signals: (i) priming, which is provided by TLRs, NOD2, and TNFR1/2 mediated by MYD88, leading to NFκB-mediated expression of inflammasome genes (pro-IL-1β and NLRP3). Pro-IL-18 is constitutively expressed in the cell. (ii) PAMPs or DAMPs (phagosomal rupture) trigger NLRP3, ASC, and caspase-1 assembly to the inflammasome, which leads to proteolytic cleavage of pro-IL-1β and pro-IL-18 for secretion and the induction of pyroptosis [98]. The non-canonical inflammasome pathway is defined by its requirement of caspase-4/-5 in human macrophages. Thus far, this way of inflammasome activation has mainly been described to be triggered by lipopolysaccharide (LPS) of Gram-negative bacteria. Unpublished data by Ott and co-workers indicated that putatively secreted corynebacterial proteins bind an unknown receptor, leading to induction of an alternative NFκB-pathway and expression of inflammasome genes. Intracellular corynebacterial effector proteins seem to induce caspase-4/-5 inflammasome assembly, resulting in proteolytic cleavage of gasdermin D, pore assembly, and pyroptosis. In this case, there is no IL-1β secretion, but intracellular IL-1R2-bound pro-IL-1α to is processed to IL-1α by caspase-5, leading to passive efflux of IL-1α through GSDMD pores ([99], Ott et al., unpublished; figure created with BioRender.com).

In addition to caspase-1, caspase-4 and -5 may provide an alternative mechanism of inflammasome activation by some *Corynebacterium* species, which also leads to cleavage of gasdermin D followed by pore formation and pyroptosis (Figure 7). Caspase-1-dependent inflammasome activation is known as canonical way, and the caspase-4/5-dependent process is termed non-canonical. The term “canonical pathway” refers to idealized or generalized pathways describing common properties of a particular signaling module or pathway, while “non-canonical pathway” refers to a less known or alternative pathway [92,93]. It is remarkable that some *C. diphtheriae* strains induce both pathways and others do not, which has to be further characterized. It is also worth to mention that caspase-4/5-dependent inflammasome activation and pyroptosis do not lead to IL-1β secretion, since pro-IL-1β can only be processed to active IL-1 β by caspase-1 [94]. The complex process of canonical and non-canonical inflammasome activation, which seems to be induced by corynebacteria is depicted in Figure 8. The involvement of caspase-4/5 and IL-1α in response to *C. diphtheriae* infection is hypothetical and needs to be clarified in future experiments.

## 7. Conclusions

The interaction of *C. diphtheriae* with host cells is much more complex than initially expected when *C. diphtheriae* was considered as extracellular pathogen. Various clinical isolates were investigated in host–pathogen interaction studies thus far, and their effects on the host cell differ dramatically. One reason is a wide range of (multifunctional) virulence factors, often acquired by horizontal gene transfer, contributing to various extents to adhesion, invasion, and cell damage. In addition to further studies with respect to the characterization of *C. diphtheriae* virulence factors, a major direction of future studies may include identification and characterization of receptors on the host cell and the host signaling pathways activated by *C. diphtheriae*. A more general question, which may be addressed, is which ecological benefit invasive infections may have for the bacterium.

## Figures and Tables

**Table 1:** Worldwide cases of diphtheria and vaccination coverage 2009 to 2019.

Year	Number of Diphtheria Cases	Third Dose DTP Vaccination Coverage (%)
2009	4349	89.06
2010	4603	89.48
2011	5626	89.90
2012	4490	90.33
2013	4680	89.54
2014	7774	89.84
2015	4535	89.03
2016	7102	89.16
2017	8819	88.77
2018	16,611	89.22
2019	22,986	89.70

**Table 2:** *C. diphtheriae* virulence factors and their putative human and murine receptors.

*C. diphtheriae* Component	Interacting Host Cell Receptor	Function and Experimental System	Reference
CdiLAM and other glycolipids	C-type lectin receptor Mincle,	adhesion to human epithelial cells agglutination of human erythrocytes	[45,57,58]
	TLR2	Mincle activation in primary mouse macrophages	
Pili	laminin	adherence to human epithelial cells colonization of *C. elegans*	[34,36,38,59]
CpG methylated DNA	TLR9	activation of TLR9 in human macrophages	[58]
DIP0733	fibrinogen fibronectin collagen	agglutination of human erythrocytes adherence to human epithelial cells invasion of human epithelial cells collagen and fibrinogen-binding induction of apoptosis colonization of *C. elegans* lethal to *Galleria.mellonella*	[40,51,60]
DIP1281	unknown	adherence to human epithelial cells	[42]
DIP1546	unknown	adherence to human epithelial cells colonization of *C. elegans*	[43]
DIP1621	unknown	adherence to human epithelial cells	[44]
DIP2093	fibrinogen fibronectin collagen	collagen binding adherence to human epithelial cells invasion into human epithelial cells colonization of *C. elegans* arthritis in mice	[41]
Rbp	unknown	cytotoxic effect to Vero cells (green monkey kidney cells) apoptosis and necrosis in human macrophages and epithelial cells detrimental effects to *C. elegans* and *G. mellonella*	[61]
Diphtheria toxin	HB-EGF EF-2	receptor-mediated endocytosis of the toxin in human cells ADP ribosylation and stop of protein synthesis lethal to guinea pigs	[62,63,64,65]

## Data Availability

Not applicable.
